# Thermodynamic Analysis of Climate Change

**DOI:** 10.3390/e25010072

**Published:** 2022-12-30

**Authors:** Nabil Hazzaa Swedan

**Affiliations:** Pacific Engineering PLLC, 9350 Red-Wood Rd. NE, B210, Redmond, WA 98052, USA; nabilswedan@yahoo.com or swedan@pacificengineeringpllc.com

**Keywords:** energy consumption, deforestation, surface greening, thermodynamics, Carnot cycle, psychrometry

## Abstract

The climate change assessment of the Intergovernmental Panel on Climate change is based on a radiative forcing methodology, and thermodynamic analysis of the climate does not appear to be utilized. Although equivalent to the radiative model, the thermodynamic model captures details of thermodynamic interactions among the earth’s subsystems. Carbon dioxide emission returns the net chemical energy exchanged with the climate system to the surface of the earth as heat. The heat is equal to the sum of the heat produced by fossil fuels and deforestation minus the heat of surface greening. Accordingly, trends of climate parameters are calculated. Nearly 51.40% of carbon dioxide production has been sequestered by green matter, and surface greening is approximately 3.0% per decade. Through 2020, the heat removed by surface greening has approached 12.84% of the total heat. Deforestation on the other hand has contributed nearly 22.85% of the total heat of carbon conversion to carbon dioxide. The increase in sea and average land surface air temperatures are 0.80 °C and 1.39 °C, respectively. Present annual sea level rise is nearly 3.35 mm, and the calculated reductions in the temperature and geopotential height of the lower stratosphere are about −0.66 °C and −67.24 m per decade, respectively. Unlike natural sequestration of carbon dioxide, artificial sequestration is not a photosynthetic heat sink process and does not appear to be a viable methodology for mitigating climate change.

## 1. Introduction

Chapter 8 of the Intergovernmental Panel on Climate Change (IPCC) report [1] presents in detail the radiative methodology used in assessing climate change. The methodology is based on the radiative forcing (RF) concept, available in textbooks of atmospheric physics, for example [2]. Climate agents released into the atmosphere, such as carbon dioxide, methane, nitrous oxide, and others trap long wave radiation emitted from the surface of the earth. In Table 8.2 of Chapter 8, the values of radiative forcing in W m^−2^ with respect to 1750 for climate agents are summarized. Their net contribution through 2005 was approximately equal to RF = 2.64 W m^−2^, which is equal to the total radiation imbalance at the top of the atmosphere. The climate energy balance is simplified by dT = λ RF, where dT is equal to the variation in global mean surface temperature, and λ is the climate sensitivity. If the variation in the concentration of climate agents is known, RF may be determined and the variation in the global mean surface temperature calculated. The IPCC report does not appear to provide alternative methodologies to analyze climate change. As will be demonstrated in this work, the thermodynamic methodology is more informative than the radiative forcing methodology and may provide details of thermodynamic interactions among the Earth’s subsystems.

Living matter instinctively multiplies and increases in size when conditions are favorable [3], and humans are not an exception. The natural laws applicable to the growth of plants and organisms apply to the world population, as well [4]. Deforestation and farming of the cleared land have provided carrying capacity for people. Deforestation is the opposite process of photosynthesis; it produces chemical energy. Fossil fuels are also consumed by a steadily growing population. These forms of energy exchanged in the climate system are returned to the surface of the Earth because carbon dioxide is produced in the process. It possesses a chemical potential that is equivalent to a potential energy applied onto the atmosphere as demonstrated in this work. The potential energy transfers the chemical energy exchanged to the surface of the earth as heat and changes the climate. Accordingly, trends of climate parameters are calculated for each period of time under consideration and tabulated in Table 1. Each column of the table represents climate heat and mass balance of the earth’s ocean, glaciers, atmosphere, living green matter, and fossil fuels consumed. Additionally, the table presents observed trends of climate parameters for comparison with the calculated values, and an overall agreement appears to exist. This indicates that the laws of conservation of energy and mass may be used to assess changes in climate, and a wealth of climate-related information and understanding may become possible, a potential advantage over the radiative forcing methodology. The submitted work includes theory sections encompassing atmosphere as a thermodynamic cycle, data, calculation method and error analysis, sample calculations, and discussion and conclusions. Because of the interdisciplinary nature of this work, a section entitled “Symbols and Abbreviations” is included. In this section, the meanings of the symbols used are explained.

## 2. Theory and Analysis

The objective of this section is to derive a correlation between carbon dioxide emitted into the atmosphere and the net heat gained by the earth’s subsystems. The subsystems in consideration are atmosphere, ocean, glaciers, land, and surface biomass. The heat gained by land whether from climate or variation in geothermal heat flow is typically neglected, for example [1]. This, of course, implies an error whose value may be equal or less than 6%, as explained in the discussion section. For simplicity, the heat gained by land will not be considered. The theory section is divided into subsections and the relevant equations published elsewhere are briefly explained and summarized in these subsections.

### 2.1. Heat and Mass Balance

**Figure 1:**
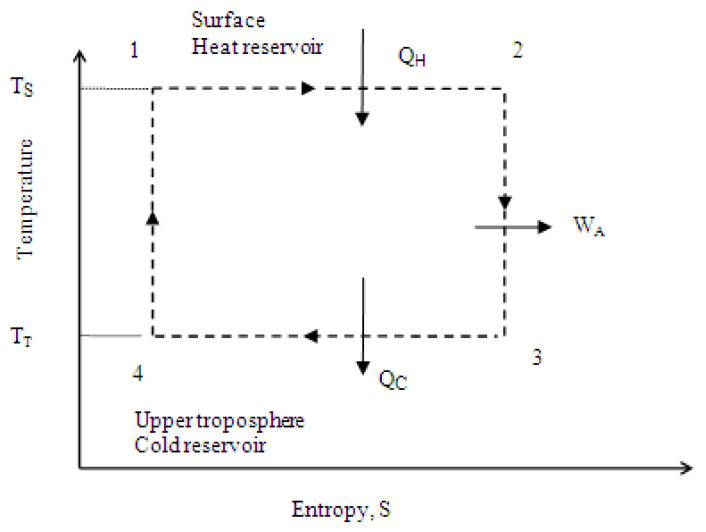
A schematic representation of the atmosphere as an ideal Carnot heat engine cycle. Q_H_ = heat supply by the heat reservoir, surface water, J; Q_C_ = heat rejected to the cold reservoir, upper troposphere, J; W_A_ = work produced by the atmosphere, J; T_S_ = average surface temperature, °K; and T_T_ = average temperature of the upper troposphere, °K.

Carnot cycle representation of atmospheric processes has been used for decades [5,6]. The medium of heat transfer of the thermodynamic cycle is moist air of the lower atmosphere. Figure 1 is a schematic representation of the atmosphere as an ideal Carnot heat engine cycle where variation in the content of carbon dioxide in the atmosphere is negligible. At average conditions, the Earth’s surface water is the heat reservoir at average surface temperature, T_S_, and the atmosphere is the cold reservoir. Water vapor condenses in the upper troposphere, and, at the tropopause, the vapor condenses completely. Average temperature of the upper tropopause, T_T_, may therefore be assumed to be equal to the temperature of the cold reservoir. The medium of heat transfer, moist air, removes surface heat Q_H_ by evaporating water. This transformation is represented by isothermal air expansion between points 1 and 2 at surface temperature T_S_. The air along with water vapor then adiabatically expands in the lower atmosphere from point 2 at the surface to point 3 in the upper troposphere, and the work, W_A_, is produced. This work raises the air mass against gravity and maintains air circulation. Under pressure of the upper atmosphere, water vapor condenses in the upper troposphere. This transformation is represented by isothermal compression from point 3 to point 4, and the heat, Q_C_, is rejected to the colder atmosphere. The dry and cold air and condensed water then return to the surface by gravity from point 4 located in the upper troposphere to point 1 at the surface, and the thermodynamic cycle repeats. At average conditions, Q_H_ = Q_C_ + W_A_.

Human population consumes energy, which has been mostly fossil fuel-based energy, and carbon dioxide is released in the process. Deforestation releases carbon dioxide as well. Surface greening sequester some of the carbon dioxide, and the net amount of carbon dioxide interacts with water vapor in the atmosphere in accordance with Equation (8) of [7]

dn_CO2_ µ_CO2_ + dn_H2O_ µ_H2O_ = 0 (1)

where;

dn_CO2_ = Variation in the number of moles of carbon dioxide in the atmosphere, mol.
µ_CO2_ = Chemical potential of carbon dioxide, −393.14 kJ mol^−1^.
dn_H2O_ = Variation in the number of moles of water vapor in the atmosphere, mol.
µ_H2O_ = Chemical potential of water vapor, 43.97 kJ mol^−1^.

Derivation of Equation (1) is lengthy, and it is available in [7]. Fundamentally, the source equation is equation 4–125 of [8], quoted as follows:

*“d(nG) = −(nS) dT + (nV) dP + Σμ_i_ dn_i_* (2)

*μ_i_= [∂(nH)/∂n_i_] _nS, P, nj_* (3)

*[∂(nG)/∂T]_P_ = −nS* (4)

*where;*

*G = Molar Gibbs Function of the thermodynamic system, J mol^−1^.*
*S = Molar entropy of the thermodynamic system, J mol^−1^ °K^−1^.*
*n = Total number of moles in the system.*
*n_i_ = Total number of moles of the chemical specie, i, present in the system.*
*n_j_ = Number of moles of all species other than specie, i, to be held as constant.*
*T = Temperature of the thermodynamic system, °K.*
*V = Molar volume of the thermodynamic system, m^3^ mol^−1^.*
*P = Pressure of the thermodynamic system, Pa.*
*μ_i_ = Chemical potential of the i-th specie, J mol^−1^.*
*H = Molar enthalpy of the thermodynamic system, J mol^−1^.”*

If the atmosphere is considered as a thermodynamic system, the surrounding outer space exerts a negligible pressure that may be assumed to be constant. Equation (4) gives [∂(nG)/∂T]_P_ = d(nG)/dT = −nS, and d(nG) = −(nS) dT. For the atmospheric air having carbon dioxide and water vapor as chemical species, Equations (2)–(4) reduce to Equation (1). The chemical potentials of carbon dioxide and water vapor are equal to the heat of carbon combustion and latent heat of water evaporation, respectively. The chemical potentials of greenhouse gases other than carbon dioxide have been neglected in Equation (1). They have short life and negligible variation in the number of moles compared with carbon dioxide. For atmospheric air mass of 5.18 × 10^18^ kg and air molecular weight of 28.8 [9], Equation (1) yields

dn_H2O_ µ_H2O_ = −dn_CO2_ µ_CO2_ = 7.07 × 10^19^ dppmv_CO2_ (5)

where;

dppmv_CO2_ = Variation in the concentration of carbon dioxide in the atmosphere in parts per million by volume, ppmv.

Referring to Figure 1, if the atmosphere is considered as a thermodynamic system, at average conditions, the energy balance follows

Q_H_ = Q_C_ + W_A_ (6)

where;

Q_H_ = Heat supply by the heat reservoir, the surface, at average surface temperature, J.
Q_C_ = Heat rejected to the cold reservoir, the atmosphere, at average temperature of the upper troposphere, J.
W_A_ = Work produced by the atmosphere, J.

From Equation (5), if the concentration of carbon dioxide increases in the atmosphere, water vapor forms in the atmosphere. Or, some of surface evaporation remains uncondensed in the atmosphere. The atmosphere thus loses energy that is equal to the latent heat of water vapor, dn_H2O_ µ_H2O_. This deviation from average conditions may be represented by the following heat balance:

Q_H_ = Q_C_ − dn_H2O_ µ_H2O_ + W′_A_ (7)

where;

W′_A_ = Work produced by the atmosphere after an amount of carbon dioxide that is equal to dn_CO2_ has been added to the atmosphere, J.

Subtraction of Equation (6) from Equation (7) and keeping in mind Equation (1) gives

dW_A_ = W′_A_ − W_A_ = dn_H2O_ µ_H2O_ = −dn_CO2_ µ_CO2_ (8)

dW_A_ = −dn_CO2_ µ_CO2_ (9)

**Figure 2:**
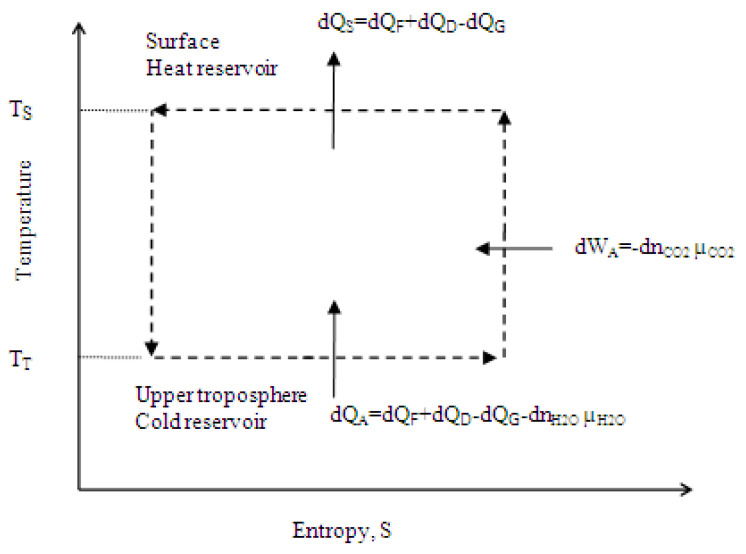
A schematic representation of the present carbon cycle as a reverse ideal Carnot heat engine cycle. dW_A_ = work of the force of gravity applied on the atmosphere, J; dQ_A_ = heat transferred from the atmosphere to the surface of the earth, J; dQ_S_ = total energy transferred to the surface of the earth, J; T_S_ = average surface temperature, °K; T_T_ = average temperature of the upper tropopause, °K; dQ_F_ = heat of combustion of fossil fuels, J; dQ_D_ = heat of deforestation, J; dQ_G_ = heat of surface greening, J; dn_CO2_ = variation in the number of moles of carbon dioxide in the atmosphere, mol; µ_CO2_ = chemical potential of carbon dioxide, kJ mol^−1^; dn_H2O_ = variation in the number of moles of water vapor in the atmosphere, mol; µ_H2O_ = chemical potential of water vapor, kJ mol^−1^.

Equation (9) expresses equality between variation in the potential energy of the atmosphere and the opposite sign of variation in the chemical potential of carbon dioxide in the atmosphere. If the concentration of carbon dioxide increases, an external work of the force of gravity thus applies onto the atmosphere that is equal to dW_A_. This work slightly reverses the natural thermodynamic cycle of the atmosphere, as shown in Figure 2. If now all of the earth subsystems (ocean, glaciers, land, green matter, and the surrounding atmosphere) are considered as a thermodynamic “climate” system, the heat exchanged with the climate system includes heat of combustion of fossil fuels, heat of deforestation, and heat removed by green matter. Carbon dioxide is exchanged, as well, and simultaneously, water vapor forms in accordance with Equation (1). Therefore, an amount of heat that is equal to the latent heat of water evaporation, dn_H2O_ µ_H2O,_ is removed from the system. Based on [8], the first law of thermodynamics for the system in consideration follows:

dQ_F_ + dQ_D_ − dQ_G_ − dn_H2O_ µ_H2O_ = dU + dW (10)

dU = dH − d (PV)(11)

where;

dQ_F_ = Variation in the chemical energy of fossil fuels, J.
dQ_D_ = Variation in the chemical energy of deforestation, J.
dQ_G_ = Variation in the chemical energy of living green matter, J.
dU = Variation in the internal energy of the climate system, J.
dW = Variation in the mechanical energy or potential energy of the climate system, J.
dH = Variation in the enthalpy of the climate system, J.
P = Pressure applied on the system, Pa.
V = Volume of the system, m^3^.

The system as defined is surrounded by an empty outer space and P = 0. Therefore, Equation (11) gives dU = dH. Variation in the enthalpy dH is equal to the sum of variation in the heat content of ocean and glaciers, dQ_S_, and variation in the enthalpy of the atmosphere, dH_A_. The heat gained by land is neglected, as discussed earlier. Because the work, dW, is exchanged only with the atmosphere as potential energy, dW is equal to the variation in the potential energy of the atmosphere, dW_A_. Additionally, because the thermodynamic cycle reverses, this work exchanged, dW_A,_ is not produced by the atmosphere; it is applied on the atmosphere instead by the force of gravity and must have an opposite sign of W_A_. Therefore, Equations (10) and (11) yield:

dQ_F_ + dQ_D_ − dQ_G_ − dn_H2O_ µ_H2O_ = dQ_S_ + dH_A_ − dW_A_ (12)

where;

dQ_S_ = Variation in the heat content of ocean and glaciers, J.
dH_A_ = Variation in the enthalpy of the atmosphere, J.

The heat, dn_H2O_ µ_H2O,_ that forms in the atmosphere is latent in nature; it does not add sensible heat to the atmosphere. It adds only an infinitesimal amount of uncondensed water vapor, or gas, having the same temperature as the atmosphere. Therefore, dH_A_ ≈ 0. Because −dn_H2O_ µ_H2O_ and −dW_A_ are equal based on Equation (8), these two terms cancel out, and Equation (12) simplifies to:

dQ_S_ = dQ_F_ + dQ_D_ − dQ_G_ (13)

Equation (13) indicates that variation in the heat content of the ocean and glaciers, dQ_S_, is equal to the variation in the net chemical energy exchanged. This equality may be used to generate the heat and mass balance of climate change for any period of time. Because fossil fuel combustion, deforestation, and surface greening exchange carbon dioxide with the atmosphere, the heat dQ_S_ may be obtained by knowing the net variation in the content of carbon dioxide in the atmosphere as well. As discussed earlier, when carbon dioxide is released, the natural heat engine cycle slightly reverses, as shown in Figure 2. The work exchanged, dW_A_, is equal to the variation in the potential energy of the atmosphere that is equal to 7.07 × 10^19^ dppmv_CO2_ based on Equations (5) and (9). Therefore:

dQ_S_ = dQ_F_ + dQ_D_ − dQ_G_ = dW_A_/η_A_ = 7.07 × 10^19^ dppmv_CO2_/η_A_ (14)

where;

η_A_ = Efficiency of the atmosphere considered as a Carnot heat engine cycle, dimensionless. The value of η_A_ is nearly equal to 0.17, estimated in the calculation section.

### 2.2. Surface Temperature and Sea Level Rise

The calculated net heat returned to the surface, dQ_S_, may be obtained by either Equation (13) or Equation (14). This heat is gained by the surface infinitesimally with time. Surface temperature rises and glaciers melt, and these two processes may be assumed to be in equilibrium. In the calculation method section, it is demonstrated that the heat dQ_S_ divides about equally between ocean and glaciers. Therefore, an amount of heat that is equal to dQ_S_/2 raises surface temperature as follows:

dQ_s_/2 = M_A_ C_PA_ dT_S_ (15)

E = M_A_ W_S_ (16)

dT_S_ = (dQ_S_/2) × W_S_/(Γ × C_PA_) (17)

where;

M_A_ = Mass of the circulated surface dry air, kg yr^−1^.
C_PA_ = Specific heat of air, 1000 J kg^−1^ °C^−1^.
dT_S_ = Sea surface temperature rise, which is equal to sea air temperature rise, °C.
W_S_ = Air humidity at saturation with sea water, kg water per kg dry air, dimensionless.
E = Annual evaporation, kg yr^−1^.
Γ = Annual precipitation, which is equal to annual evaporation, 4.86 × 10^17^ kg yr^−1^.

Equation (17) is obtained by eliminating M_A_ from Equations (15) and (16), and the derived Equation (17) is identical to the published Equation (4) of [10]. The calculated dT_S_ by Equation (17) is equal to sea air temperature rise. This air is saturated with sea water, and it is thus on the saturation curve of the psychrometric chart. Therefore, dT_S_ represents the increase in global air wet bulb temperature. The increase in land air temperature may be estimated as:

dT_L_ = dW_S_ L_V_/C_PA_ (18)

where;

dT_L_ = Land surface air temperature rise, °C.
L_V_ = Latent heat of water evaporation at surface conditions, 2461.3 kJ kg^−1^.

The calculated rise in the land air temperature by Equation (18) does not account for the contribution of sea air temperature rise. The average rise in the land surface air temperature is nearly equal to the weighted average value of dT_S_ and dT_L_. Sea level rise due to glaciers melting may be obtained by dividing the volume of the melted glaciers by sea area:

dh = (dQ_S_/2)/(L_F_ × 0.7 × 5.1 × 10^14^) (19)

where;

dh = Sea level rise, mm.
L_F_ = Latent heat of ice melting, 334 kJ kg^−1^.

The values 0.7 and 5.1 × 10^14^ in the denominator of Equation (19) are the area ratio between surface water and the total surface area of the earth, dimensionless, and the total surface area of the earth, m^2^.

### 2.3. Chemical Energy of Fossil Fuels, dQ_F_

The chemical energy of fossil fuels is nearly equal to the majority of the cumulative energy production by the world population. The United States Energy Information Administration [11] has published a series of reports relative to international energy consumption titled “International Energy Outlook.” For example, Figure 1 and Figure 2 on page 8 of International Energy Outlook 2016 presents annual energy consumption of OECD countries and non-OECD countries. The total energy consumed is equal to the sum of the two. In 1990 and 2010, the energy consumed by the world in those years was 3.70 × 10^20^ J and 5.68 × 10^20^ J, respectively. International Energy Outlook 2020 gives energy consumption of 6.3 × 10^20^ J for 2019. Before 1990, annual energy consumption data are unavailable, and linearity is assumed. By knowing the annual energy production, the cumulative chemical energy of fossil fuels may be calculated.

### 2.4. Chemical Energy of Deforestation, dQ_D_

The value of the heat of deforestation dQ_D_ may be computed by using the published Equation (11) of [4], which is quoted below

*“Q_D_ = −2.22 × 10^22^ d [(1/η* *_max_) Exp(η* *_max_ n) − η n^2^/2 − 1/(η* *_max_)], where 2.22 × 10^22^ is equal to the initial biomass inventory, J; d, annual deforestation fraction, dimensionless; η* *_max_, maximum value of seasonal efficiency of photosynthesis, dimensionless; η, average value of seasonal efficiency of photosynthesis, dimensionless; n, number of deforestation years”*

It should be noted that the value of the heat of deforestation calculated by this equation is based on an initial terrestrial biomass of 2.22 × 10^22^ J for the Industrial Era. To account for earlier deforestation since farming was invented, the initial biomass has been increased to 2.41 × 10^22^ J, as discussed in the data section. The general form of the heat of deforestation is:

dQ_D_ = −Q_G0_ × d [(1/η_max_) Exp(η_max_ n) − η n^2^/2 − 1/η_max_] (20)

where;

dQ_D_ = Chemical energy of deforestation, J.
Q_G0_ = Initial terrestrial biomass inventory, J.
d = Annual deforestation fraction, dimensionless.
η = Average value of the seasonal efficiency of photosynthesis, dimensionless.
η_max_ = Maximum value of the seasonal efficiency of photosynthesis, dimensionless.
n = Number of deforestation years, yr.

### 2.5. Chemical Energy of Surface Greening, dQ_G_

Surface greening is equal to the trend in the efficiency of seasonal photosynthesis with time. By knowing the initial biomass inventory, the heat removed from the surface may be calculated. In analogy with Equation (17) of [10], the chemical energy of surface greening may be calculated as follows:

Q_G_ = Q_G0_ Exp [η_max_ (t − t_0_)] (21)

Q_G_ = Q_G0_ Exp [η (t − t_0_)] (22)

dQ_G_ = Q_G0_ Exp [η (t − t_0_)] × dη × (t − t_0_) (23)

where;

Q_G_ = Biomass inventory at a time t, J.
Q_G0_ = Initial biomass inventory at an initial time t_0_, J.
dQ_G_ = Chemical energy of surface greening, J.
t − t_0_ = Period of time in consideration, yr.
dη = Annual increase in photosynthesis efficiency, which is equal to annual surface greening fraction, dimensionless.

Equation (21), which is the published Equation (17) of [10], has been used to calculate seasonal growth of a single tree and field crop. The concentration of carbon dioxide in the atmosphere varies seasonally; however, at the end of the year, the concentration of carbon dioxide does not change because seasonal decay cancels out seasonal growth of green matter. Surface greening is negligible, regardless of the time factor (t − t_0_). The scenario is different for the present carbon cycle because the concentration of carbon dioxide in the atmosphere increases every year. The annual average value of photosynthesis efficiency, η, is used instead of its seasonal maximum value, η_max,_ as shown in Equation (22). Differentiation of this equation yields Equation (23). The variable in Equation (23) is the efficiency of photosynthesis, η, and it is increasing because the concentration of carbon dioxide is increasing. The value of the annual surface greening fraction, dη, may be obtained from the published Equation (13) of [10], which is quoted as follows:

*“% Greening = 100 × (dppmv/2)/ppmv, where dppmv is annual increase in the concentration of carbon dioxide in the atmosphere in parts per million by volume, ppmv; ppmv, is average concentration of carbon dioxide in the atmosphere, ppmv.”*

Therefore, the annual surface greening fraction dη for Equation (23) is equal to:

dη = (dppmv_CO2_/2)/ppmv_CO2_ (24)

where;

dppmv_CO2_ = Annual increase in the concentration of carbon dioxide in the atmosphere, ppmv.
ppmv_CO2_ = Average concentration of carbon dioxide in the atmosphere, ppmv.

## 3. Data

The data used for this work are available online and in published literature. A basic requirement for this work is establishing a reference state of climate conditions. The report [1] considers the year 1750 as the beginning of the industrial era and the present warming trend, and it is thus assumed as a reference year. For the reference year, carbon dioxide content in the atmosphere and average surface temperature are 280 ppmv and 13.7 °C, respectively. The value of the efficiency of seasonal photosynthesis for 1750 is approximately 0.0067. It is calculated in the sample calculations using the psychrometric chart and the observed land air and sea temperature trends provided by [1]. Sea water temperature of 15.5 °C, slightly less than present sea water temperature, is used in the calculations. Annual energy consumptions are provided by [11], starting in 1990. For lack of annual energy consumption data between 1750 and 1990, the annual energy consumption is assumed to be linear with time for this period with a negligible value in 1750. The values of annual energy consumption are tabulated in line 1 of Table 1. Annual deforestation fractions are required for calculating the effects of deforestation and surface greening. They are obtained from [4] and tabulated in line 2 of Table 1. Because carbon dioxide is a reactant of photosynthesis chemical reaction, its concentration in the atmosphere is fundamental for analyzing climate change. The observed values, line 3 of Table 1, are obtained from Mauna Loa CO2 annual mean data [12]. The textbook [9] describe physics of the atmosphere. This reference, along with [13], provide physical, thermodynamic, and geometrical parameters of Earth’s surface, ocean, atmosphere, air, water, and water vapor. They are used as sources of data for the calculations. Reference [14] provides the air psychrometric data and relationships used.

The size of green matter before farming was invented is important for calculation of deforestation and terrestrial and aquatic greening. The world population size is correlated with deforestation and farming of the cleared land area [4]. References [4,10] estimated terrestrial biomass of the Industrial Era to be nearly equal to 2.22 × 10^22^ J based on 50% deforestation since that time. Terrestrial biomass before farming was invented must be greater than that calculated for the Industrial Era. The world population approached 1 billion during the Industrial Era, compared with the present population of 7 billion [15]. Therefore, the initial inventory of terrestrial biomass before land clearing may be (1/6) × 0.5 × 2.22 × 10^22^ = 1.85 × 10^21^ J greater than the estimated value for the Industrial Era. The biomass of terrestrial photosynthesis was thus nearly equal to 2.41 × 10^22^ J. In the methodology section, it is demonstrated that aquatic and terrestrial biomasses increased equally with time and that biomasses of aquatic and terrestrial photosynthesis were nearly equal before human activities.

For comparison with calculation results, observations on changes of the surface, ocean, and atmosphere provided by [1] are used. They are available in Chapters 2, 8, and 13 of this reference [1]. The authors of [16,17] observed greening of the surface, including semiarid areas, estimated between 2.08% and 4.55% per decade. The geopotential height of the upper atmosphere has been decreasing [18], and greenhouse gases are not excluded as a cause. Based on this paper [18], a reduction trend of 100 m to 340 m has been observed between 1969 and 1998, and this decrease may be used for comparison with the calculations. The publication [19] reveals that, since 1958, the surface has sequestered 52% of the total carbon dioxide produced. Additionally, the percent of the carbon sequestered by the surface between 1850 and present is nearly 55% of the total.

## 4. Calculation Method and Error Estimation

The chemical energy returned to the surface as heat by the atmosphere is exchanged with ocean surface, glaciers, atmosphere, and terrestrial and aquatic photosynthesis. It is thus important to estimate the share of each subsystem of the total heat. As discussed in the theory section, the medium of heat transfer of the Carnot heat engine of the atmosphere is moist air of the lower atmosphere. Dry air is an inert gas and does not change phase in a full thermodynamic cycle. Therefore, water evaporation and condensation processes exchange heat between the atmosphere and the surface of the earth. For the present climate change, water vapor condenses under the compression force of the atmosphere and transfers the heat to the surface, Figure 2. It may be demonstrated that the heat returned by the atmosphere to the surface of the earth, dQ_S_ of Equation (13), divides about equally between ocean and glaciers. The heat is in the form of latent heat of a slightly compressed water vapor. It condenses on ocean surface and glaciers. If the glaciers receive more heat than the ocean, the glaciers discharge more cold water as glacier melt. The cooler ocean surface now condenses more water vapor and increases the ocean’s share of heat. Assuming that the heat gained by the ocean, Q_O_, is greater than the heat gained by the glaciers, Q_GL_. The ocean surface temperature would rise by an additional amount that is equal to:

dT_O_ = (Q_O_ − Q_GL_)/(M C_PW_) (25)

where;

dT_O_ = Additional rise in ocean surface temperature, °K.
Q_O_ = Heat gained by the ocean, J.
Q_GL_ = Heat gained by the glaciers, J.
M = Mass of ocean surface water engaged in the heat transfer, kg.
C_PW_ = Specific heat of ocean water, J kg °k^−1^.

The additional rise in ocean surface temperature by dT_O_ decrease the rate of water vapor condensation on ocean surface:

dQ_O_/dt = −U_T_ A dT_O_ (26)

dQ_O_/dt = −U_T_ A(Q_O_ − Q_GL_)/(M C_PW_) (27)

dQ_O_/dt + [U_T_ A/(M C_PW_)] Q_O_ = [U_T_ A/(M C_PW_)] Q_GL_ (28)

where;

t = Time, s.
U_T_ = Heat transfer coefficient between atmosphere and ocean surface, J s^−1^ m^−2^ °K^−1^.
A = Heat transfer surface area between ocean and atmosphere, m^2^.

Equation (28) is a differential equation of the first order whose solution follows

Q_O_ = Exp [−t U_T_ A/(M C_PW_)] + Q_GL_ (29)

For a period of time large enough, Q_O_ = Q_GL_, and the heat returned by the atmosphere is divided about equally between ocean and glaciers.

Water vapor formation, dn_H2O_ µ_H2O_, due to carbon dioxide emission occurs in the lower atmosphere. As a result, this latent heat is denied to the upper atmosphere. The heat lost by the upper atmosphere, dn_H2O_ µ_H2O_, decreases the enthalpy and potential energy of the upper atmosphere equally with time. If the upper atmosphere is considered as a thermodynamic system, the lower atmosphere and outer space are the surroundings. The decrease in the temperature of the upper atmosphere is of the order of 1.2 × 10^−5^ °C hr^−1^. It is an infinitesimal decrease compared with practical applications. The decrease in the energy of the upper atmosphere may be considered as thermodynamic transformations displaced differentially apart. They are virtually frictionless and reversible. Consequently, variation in the total entropy of the system and surroundings approaches zero [8]. Because the outer space is empty and at a temperature near zero absolute, the variation of its entropy may be neglected based on the third law of thermodynamics. The lower atmosphere is adiabatic [9], and variation in the entropy of the lower atmosphere may be neglected as well. Therefore, for the upper atmosphere:

dS_U_ ≈ 0 (30)

where;

S_U_ = Entropy of the upper atmosphere, J °K^−1^.

From Equation (30), dS_U_ = dQ_U_/T_U_ = 0. dQ_U_ and T_U_ are the heat lost by the upper atmosphere in Joules and average temperature of the upper atmosphere in °K, respectively. Similar thermodynamic analysis to that presented in the theory section for the upper atmosphere (instead of the entire atmosphere) gives dQ_U_ = dH_U_ + dW_U_. Therefore, (dH_U_ + dW_U_)/T_U_ = 0, and dH_U_ = −dW_U_, where dH_U_ and dW_U_ are the decrease in the enthalpy and potential energy of the upper atmosphere in Joules, respectively. They decrease about equally with time and differ only by a conventional negative sign.

It may be demonstrated that the aquatic and terrestrial photosynthesis produced about equal biomasses before humans became influential on the climate. Photosynthesis is a chemical reaction where green matter converts carbon dioxide and water into glucose and oxygen. Therefore, the rate of growth of aquatic photosynthesis can exceed the global average only if the concentration of carbon dioxide in the atmospheric air above sea water increases more than average, which is an unrealistic scenario because the atmospheric air is reasonably mixed. The growth rates of aquatic and terrestrial green matter may have thus been equal at all times when the concentration of carbon dioxide in the atmosphere decreased in the distant past. Or, dη_GA_/dt = dη_GT_/dt, where η_GA_ and η_GT_ are efficiencies of aquatic and terrestrial photosynthesis, respectively, and t is the time. Integration of this equation gives η_GA_ = η_GT_ + (η_GA0_ − η_GT0_), where η_GA0_ and η_GT0_ are the initial efficiency values of the aquatic and terrestrial photosynthesis, respectively. The difference (η_GA0_ − η_GT0_) is infinitesimal compared with η_GA_ or η_GT_ and may be discarded. Consequently, η_GA_ ≈ η_GT_, and the efficiencies of aquatic and terrestrial photosynthesis are about equal. Therefore, dQ_GA_/Q_GA_ ≈ dQ_GT_/Q_GT_, where Q_GA_ and Q_GT_ are aquatic and terrestrial biomasses in Joules, respectively. The last equality may be integrated between initial aquatic and terrestrial biomasses and Q_GA_ and Q_GT_, keeping in mind that the initial biomasses were small and negligible, and Q_GA_ ≈ Q_GT_. The aquatic and terrestrial biomasses may thus be assumed to be equal before farming was invented.

Accordingly, the cumulative chemical energies of global energy consumption, deforestation, and surface greening have been tabulated in lines 7, 8, and 9 of Table 1. The net heat returned to the surface is presented in line 10 in accordance with Equation (13). The share of heat for each of the earth’s subsystems is then calculated per the above discussion, and trends of climate parameters determined. Equation (13) is the fundamental equation for Table 1 preparation, and calculation error may be analyzed. For clarity, q_S_, q_F_, q_D_, and q_G_ will be used instead of dQ_S_, dQ_F_, dQ_D_, and dQ_G_. Because these variables are dependent on deforestation as discussed in the introduction section, the total error of Equation (13) follows:

Δq_S_ = Δq_F_ + Δq_D_ + Δq_G_ (31)

where Δq_S_, Δq_F_, Δq_D_, and Δq_G_ are absolute values of the errors of q_S_, q_F_, q_D,_ and q_G,_ respectively, in Joules. In terms of relative error, Δq_S_/q_S_ = (Δq_F_/q_F_) q_F_/q_S_ + (Δq_D_/q_D_) q_D_/q_S_ + (Δq_G_/q_G_) q_G_/q_S_ and:

ε_QS_ = ε_QF_ q_F_/q_S_ + ε_QD_ q_D_/q_S_ + ε_QG_ q_G_/q_S_ (32)

where;

ε_QS_ = Δq_S_/q_S_, relative error of the calculated heat exchanged with the surface q_S_, J.
ε_QF_ = Δq_F_/q_F_, relative error of the world energy consumption data q_F_, J.
ε_QD_ = Δq_D_/q_D_, relative error of the calculated heat of deforestation q_D_, J.
ε_QG_ = Δq_G_/q_G_, relative error of the calculated heat of surface greening q_G_, J.

Equation (20) has annual deforestation fraction, d, as an independent parameter. The value of ε_QD_ is therefore equal to Δd/d, where Δd is the absolute value of the error in the measured annual deforestation fraction, dimensionless. The value of ε_QG_ may be obtained from Equation (23), and it is equal to n Δη, where n is the number of years and Δη is the absolute value of the error of the calculated photosynthesis efficiency. It is correlated with the error of chemical analysis of carbon dioxide in the atmosphere, which may be obtained from Equation (24), Δη = ΔCO2/(2 ppmv_CO2_). ΔCO2 is equal to the absolute value of the error of carbon dioxide chemical analysis, ppmv. Substituting these values in Equation (32) gives:

ε_QS_ = ε_QF_ q_F_/q_S_ + (Δd/d) q_D_/q_S_ + [n ΔCO2/(2 ppmv_CO2_)] q_G_/q_S_ (33)

Equation (33) summarizes the contributors of the calculation error. They are data of energy consumption, annual deforestation fraction, chemical analysis of carbon dioxide, and the number of years for which the calculations are conducted. The error in the chemical analysis of carbon dioxide ΔCO2 is small, less than 0.1 ppmv [20]. From Table 1, lines 9 and 10, the present ratio between the heat of surface greening and the total heat returned to the surface q_G_/q_S_ = 1 × 10^22^/6.79 × 10^22^ = 0.15. Should the calculations be conducted on an annual basis, n = 1, the last term of Equation (33) may be neglected. From Table 1, lines 7 and 10 and lines 8 and 10, the heat ratios q_F_/q_S_ and q_D_/q_S_ are 0.89 and 0.26, respectively, and ε_QS_ ≈ 0.89ε_QF_ + 0.26ε_QD_, Equation (33). In the event that deforestation decreases substantially, ε_QS_ ≈ 0.86ε_QF_. Referring to Table 1, the period of time between 1750 and 1850 had mainly q_F_ and q_D_, and ε_QS_ ≈ 0.57ε_QF_ + 0.53ε_QD_. Between 1850 and 1960, ε_QS_ ≈ 0.82ε_QF_ + 0.28ε_QD_. However, these periods contributed 20% and 47% of the total heat, respectively. Therefore, the weighted average error for the entire period between 1750 and 2020 is ε_QS_ ≈ 0.79ε_QF_ + 0.32ε_QD_. Uncertainty of ±30% of the annual deforestation fraction and ±10% of the energy consumption may thus produce an error of ±17.50%.

Calculation error of Equation (14) depends principally on the value of the efficiency of the atmosphere η_A_, which is equal to η_A_ = 1 − T_T_/T_S_, where T_T_ is the average temperature of the upper troposphere, °K, and T_S_ is surface temperature, °K. Therefore:

ε_ηA_ = Δη_A_/η_A_ ≈ ΔT_T_/(T_S_ − T_T_) (34)

where;

ε_ηA_ = Relative error of the calculated efficiency of the atmosphere as Carnot heat engine, dimensionless.
T_T_ = Average temperature of the upper troposphere, °K.
T_S_ = Surface temperature, °K.
ΔT_T_ = Absolute value of the error or uncertainty in T_T_, °K.

The error or uncertainty in ΔT_T_ would be equal to the difference between the value of T_T_ suggested by the U.S. Standard Atmosphere or equal atmosphere and the actual value of T_T_ at nearly 40° latitude.

## 5. Sample Calculations

The initial photosynthesis efficiency, η_0_, for the year 1750 may be calculated from observed trends in land air and marine temperatures. The air above sea water may be assumed to be at saturation with sea surface water. The observed rise in marine temperature is thus equal to the rise in sea air temperature. This rise is equal to the rise in global air wet bulb temperature. For average land air and marine temperature rise of 1.4 °C and 0.75 °C, respectively [1], the rise in global mean surface air temperature is nearly equal to 0.7 × 0.75 + 0.3 × 1.4 = 0.95 °C. Where the figures 0.7 and 0.3 are sea and land surface area ratios, respectively. Since 1750, air dry bulb temperature T_db_ increased from of 13.7 °C (286.86 °K) to 13.7 + 0.95 = 14.65 °C. At 60% relative humidity, the present average global wet bulb temperature is 10.6 °C. The air wet bulb temperature for 1750 was thus equal to T_wb_ = 10.6−0.75 = 9.85 °C (283.01 °K). Based on [10], the efficiency of photosynthesis for 1750 is η_0_ = 1 − (T_wb_/T_db_)^0.5^ = 1 − (283.01/286.86)^0.5^ = 0.0067.

Referring to Table 1, for the period of time between 2010 and 2020, the initial terrestrial biomass Q_GT0_ = 1.69 × 10^22^ J, line 5; aquatic biomass Q_GA0_ = 2.93 × 10^22^ J, line 6; annual deforestation fraction is d = −0.0011, line 2; annual energy consumption for 2010 and 2020 are 5.68 × 10^20^ J and 7.05 × 10^20^ J, respectively, line 1; seasonal efficiency of photosynthesis, η = 0.0081, line 4; η_max_≈2 × 0.0081; sea surface temperature rise through 2010 is 0.74 °C, line 13; sea surface temperature for 2010, T_S_ = 15.5 + 0.74 = 16.24 °C and W_S_ = 0.0119 kg water per kg dry air. The period of time is ten years. Therefore, n = t − t_0_ = 10, and the calculations may be conducted between 2010 and 2020 as follows:

Chemical energy of deforestation, dQ_D_ = −1.69 × 10^22^ × −0.0011 × [1/(2 × 0.0081) × 2.718^(2 × 0.0081 × 10)^ − 0.0081 × 10^2^/2 − 1/(2 × 0.0081)] = 1.94 × 10^20^ J, Equation (20), line 8.

Chemical energy of fossil fuel between 2010 and 2020, dQ_F_ = (5.68 × 10^20^ + 7.05 × 10^20^)/2 × 10 = 6.37 × 10^21^ J, line 7.

The observed annual increase in the concentration of carbon dioxide is (414.24 − 390.1)/10 = 2.41 ppmv, line 3.

Annual surface greening fraction, dη = (2.41/2)/[(414.24 + 390.1)/2] = 0.003, Equation (24), line 11.

Terrestrial biomass greening, dQ_GT_ = 1.69 × 10^22^ × Exp [0.0081 × 10] × 0.003 × 10 = 5.50 × 10^20^ J, Equation (23).

Aquatic biomass greening, dQ_GA_ = 2.93 × 10^22^ Exp [0.0081 × 10] × 0.003 × 10 = 9.53 × 10^20^ J, Equation (23).

Chemical energy of surface greening between 2010 and 2020, dQ_G_ = dQ_GT_ + dQ_GA_ = 5.50 × 10^20^ + 9.53 × 10^20^ = 1.50 × 10^21^ J, line 9.

The amount of carbon dioxide sequestered by surface greening between 2010 and 2020 in parts per million of carbon dioxide is dppmv_CO2_ = dQ_G_/7.07 × 10^19^ = (1.50 × 10^21^)/(7.07 × 10^19^) = 21.20 ppmv, Equation (5), line 12.

The net heat to the surface dQ_S_ = dQ_F_ + dQ_D_ − dQ_G_ = 6.37 × 10^21^ + 1.94 × 10^20^ − 1.50 × 10^21^ = 5.06 × 10^21^ J, Equation (13), line 10.

Ocean share of heat = dQ_S_/2 = 2.53 × 10^21^ J; Sea air temperature rise dT_S_ = 2.53 × 10^21^ × 0.0119/(4.86 × 10 ^17^ × 1 000) = 0.060 °C, Equation (17), line 13.

Glaciers share of heat = dQ_S_/2 = 2.53 × 10^21^ J; sea level rise by glaciers melt only = 2.53 × 10^21^/(334 000 × 0.7 × 5.1 × 10^14^) = 21.23 mm, Equation (19); average annual sea level rise 21.23/10 = 2.12 mm yr^−1^, line 17.

For sea air temperature rise of 0.06 °C, variation in air humidity dW_s_ = 0.000089 kg water per kg dry air [14]; land air temperature rise, dT_L_ = 0.000089 × 2 461 300/1000 = 0.220 °C, Equation (18); average rise in land surface air temperature = 0.7 × dT_S_ + 0.3 × dT_L_ = 0.7 × 0.06 + 0.3 × 0.22 = 0.10 °C, line 15. Where the figures 0.7 and 0.3 are sea and land surface area ratios, respectively.

As discussed in the theory section, an amount of heat that is equal to dn_H2O_ µ_H2O_ = 7.07 × 10^19^ dppmv_CO2_, Equation (5), is denied to the upper atmosphere. As a result, the heat content and potential energy of the upper atmosphere decrease equally, as demonstrated in the calculation method section. The total mass of atmospheric air is M = 5.18 × 10^18^ kg, and the mass of the upper atmosphere is nearly equal to 25% of the total mass of the atmospheric air [9]. Its center of mass lies in the lower stratosphere. Therefore, average reduction in the temperature of the lower stratosphere = −(dn_H2O_ µ_H2O_/2)/[0.25 M C_PA_] = −[7.07 × 10^19^ × (414.24−390.10)/2]/[0.25 × 5.18 × 10^18^ × 1000] = −0.66 °C d^−1^, line 19. Average reduction in the height of the lower stratosphere = −(dn_H2O_ µ_H2O_/2)/[0.25 M g] = −[7.07 × 10^19^ (414.24 − 390.10)/2]/[0.25 × 5.18 × 10^18^ × 9.8] = −67.24 m d^−1^, line 21.

The average temperature of the upper tropopause, T_T_, at nearly 37.5 kPa is about 240 °K, and surface temperature, T_S_, is 288.15 °K [9]. Therefore, the efficiency of the thermodynamic cycle of the atmosphere η_A_ ≈ 1 − T_T_/T_S_ = 0.17. The total heat exchanged with the surface between 1750 and 2020 is dQ_S_ = 7.07 × 10^19^ dppmv_CO2_/η_A_ = 7.07 × 10^19^ × (414.24 − 280)/0.17 = 5.68 × 10^22^ J, Equation (14).

Through 2005, the concentration of carbon dioxide in the atmosphere increased by 99.91 ppmv. The total heat exchanged with the surface between 1750 and 2005 is equal to 7.07 × 10^19^ × 99.91/0.17 = 4.16 × 10^22^ J, Equation (14). The equivalent radiative forcing is 4.16 × 10^22^/(5.14 × 10^14^ × 3.15 × 10^7^) = 2.57 W m^−2^. The quantities in the denominator, 5.14 × 10^14^ and 3.15 × 10^7^, are the area of the surface of the earth, m^2^, and the number of seconds in one year, respectively.

Similar calculations for the periods of time of Table 1 are conducted, and the results are tabulated in the table.

## 6. Discussion and Conclusions

This work indicates that carbon dioxide emission possesses a chemical potential equivalent to a potential energy that reverses slightly the natural Carnot heat engine of the atmosphere. As a result, the atmosphere returns the net chemical energy exchanged with the climate system to the surface of the earth as heat. The value of this heat may be calculated by Equations (13) and (14). The advantage of using Equation (14) is simplicity because it requires only variation in the content of carbon dioxide in the atmosphere. Additionally, the efficiency of the atmosphere varies with latitude, and Equation (14) may be used to calculate latitudinal surface warming. Through 2005, the calculated radiative forcing by Equation (14) is 2.57 Wm^−2^, sample calculations, and the observed radiative forcing by IPCC is 2.64 Wm^−2^ as discussed in the Introduction. They are in good agreement, which confirms the equivalence between radiative and thermodynamic methodologies. Equation (13), on the other hand, is more informative, but contains more variables and error contributors. As deforestation decreases with time, Equation (13) could have accuracy equal to that of energy production data or less. Through 2020, the total heat exchanged with the surface, dQ_S_, calculated by the heat balance of Equation (13) is 6.79 × 10^22^ J, line 10 of Table 1, and the calculated heat by thermodynamic efficiency of the atmosphere, Equation (14), in the sample calculations, is 5.68 × 10^22^ J. They are within 20%.

Referring to Table 1, line 3 shows that the observed increase in the concentration of carbon dioxide in the atmosphere through 2020 is 134.24 ppmv, and the calculated amount of carbon dioxide sequestered by green matter is 141.95 ppmv, line 12. Green matter has therefore removed nearly 51.40% of the total carbon dioxide produced since the Industrial Era. The observed value is between 52% and 55%, data section. Surface greening has increased with time to nearly 3.00% per decade, line 11, and the observed value is between 2.08% and 4.55%, data section. They are in good agreement. Through 2020, surface greening has removed 12.84% of the total heat added to the climate by fossil fuels and deforestation, lines 7, 8, and 9. The cumulative contribution of deforestation to the total heat of carbon conversion to carbon dioxide is 22.85%, lines 7, 8. The calculated sea temperature rise of 0.74 °C through 2010, line 13, agrees well with the observed sea temperature rise of 0.75 °C. Average land surface air temperature rise is in agreement with observations, as well, lines 15 and 16, and the average land surface air temperature rise through 2010 is 1.28 °C, approximately 8.6% smaller than the observed 1.40 °C. The calculated annual sea level rise by considering glaciers contribution alone is 59.30% of the observed sea level rise, lines 17, 18. When, however, ocean thermal expansion of nearly 0 mm yr^−1^ in 1750 to 1.1 mm yr^−1^ in 2011 is accounted for, the observed and calculated values of annual sea level rise are in good agreement. The authors of [21] observed present annual trend of sea level rise of nearly 3.64 mm yr^−1^. The steric component of sea level rise is about 1.19 mm yr^−1^. Therefore, glaciers melting is presently contributing 67% of the total sea level rise, and this agrees reasonably with the calculated value of 59.30%. The calculated annual sea level rise for 2020 by considering the contribution of glaciers and ocean thermal expansion is nearly equal to 2.16 + 1.19 = 3.35 mm, and it is within 8% of the observed 3.64 mm. The calculated decrease in the temperature and the geopotential height of the lower stratosphere are in agreement with observations, lines 19–22. Because air density decreases with height, the temperatures of the middle and upper stratosphere decrease more than the calculated average of −0.66 °C d^−1^. The long-term implications of this cooling on the chemistry of the upper atmosphere may deserve research and further analysis.

The advantages of the thermodynamic model are illustrated in the sample calculation section and Table 1, which are comprehensive and informative. Changes in sea air temperature, land air temperature, aquatic and terrestrial biomasses, surface greening, seasonal photosynthesis efficiency, glacier mass, as well as atmospheric physical parameters may be captured, and a thorough analysis of climate change may be conducted. The table is in essence a heat and mass balance of climate change, the center of which is the equality between variation in the heat content of ocean and glaciers and variation in the chemical energy exchanged with the climate system. The validity of this equality is demonstrated by an overall agreement between calculations and observations. Additionally, it can be demonstrated that this conclusion has been valid throughout the history of the earth. Equation (13) is valid only if the variation in the number of moles of carbon dioxide in the atmosphere dn_CO2_ occurs either by photosynthesis or its reverse process of carbon conversion to carbon dioxide. If the chemical energy of fossil fuels or living green matter is not exchanged with the surroundings, surface temperature and sea level do not change with time. Therefore, artificial sequestration of carbon dioxide from the atmosphere removes carbon dioxide gas from the climate, and not necessarily heat, because it is not a photosynthetic or heat sink process. Natural sequestration of carbon dioxide, on the other hand, removes carbon dioxide and heat from the climate in the form of chemical energy stored in green matter. Although the radiative and thermodynamic analysis of climate suggest carbon dioxide sequestration to alleviate surface warming, the thermodynamic analysis reveals that the sequestration must be naturally by green matter only.

Presently, surface temperature is increasing, which may alter the temperature profile of the geothermal heat flow in the oceanic and continental lithospheres. The impact of surface temperature increases on the heat content of land and deep ocean, thus meriting consideration for a comprehensive earth related analysis. Reference [22] estimated that the land has gained nearly 5–6% of the total heat. This heat to land could originate from the climate, geothermal heat, or both. Climate contribution to land heat content should be assessed and accounted for in the calculations. Additionally, the climate system is composed of multiple sub-systems, with very dynamic tele-and inter-connections, which continually change in space, both horizontally and vertically, on the ground surface as well as in the upper troposphere. There is a corresponding flow of matter and energy that determine incessant transformations of heat from one form to another (internal, potential, kinetic, latent, and sensible). In some parts of the world, there may appear energetic synergies greatly boosting the resulting latent heat being fed into the atmosphere, such as in monsoon regions, whereas radiative, dynamic, and physical–geographical factors converge into an anomalous transport of heat generation, such as the ENSO phenomenon. The natural cycle of carbon in the climatic system is different over land and ocean areas, depending on the season, thus affecting photosynthesis rates. Dominance of low-pressure areas causes the release of carbon dioxide. Latitudinal and altitudinal upward and downward transports of air and energy from the equator to the poles within the global air-circulation model should be considered as well. Therefore, there is room for improvement, and this work should be treated as basic analysis of climate change using thermodynamics.

## Figures and Tables

**Table 1:** Calculated and observed climate parameters for the present warming trend.

	Description/Year	1750	1850	1960	1970	1980	1990	2000	2010	2020
1	Annual energy consumption	0.0	1.54 × 10^20^	3.24 × 10^20^	3.39 × 10^20^	3.55 × 10^20^	3.70 × 10^20^	4.69 × 10^20^	5.68 × 10^20^	7.05 × 10^20^
2	Annual terrestrial deforestation fraction	0.00	0.0020	0.0020	0.0020	0.0020	0.0018	0.0016	0.0014	0.0011
3	Observed CO2 in the atmosphere, ppmv	280.00	297.58	316.91	325.68	338.76	354.45	369.71	390.10	414.24
4	Efficiency of seasonal photosynthesis	0.0067	0.0069	0.0071	0.0072	0.0074	0.0075	0.0077	0.0079	0.0081
5	Terrestrial biomass, J	2.41 × 10^22^	1.99 × 10^22^	1.67 × 10^22^	1.65 × 10^22^	1.65 × 10^22^	1.65 × 10^22^	1.67 × 10^22^	1.69 × 10^22^	1.72 × 10^22^
6	Aquatic biomass, J	2.41 × 10^22^	2.48 × 10^22^	2.66 × 10^22^	2.68 × 10^22^	2.73 × 10^22^	2.79 × 10^22^	2.86 × 10^22^	2.93 × 10^22^	3.02 × 10^22^
7	Cumulative energy consumption, J	0.00	7.71 × 10^21^	3.40 × 10^22^	3.73 × 10^22^	4.08 × 10^22^	4.44 × 10^22^	4.86 × 10^22^	5.38 × 10^22^	6.01 × 10^22^
8	Cumulative heat of deforestation, J	0.00	7.22 × 10^21^	1.61 × 10^22^	1.65 × 10^22^	1.68 × 10^22^	1.71 × 10^22^	1.74 × 10^22^	1.76 × 10^22^	1.78 × 10^22^
9	Cumulative heat of surface greening, J	0.00	1.35 × 10^21^	4.27 × 10^21^	4.65 × 10^21^	5.43 × 10^21^	6.42 × 10^21^	7.46 × 10^21^	8.62 × 10^21^	1.00 × 10^22^
10	Heat transferred to the surface of the earth, J	0.00	1.36 × 10^22^	4.58 × 10^22^	4.91 × 10^22^	5.22 × 10^22^	5.51 × 10^22^	5.85 × 10^22^	6.28 × 10^22^	6.79 × 10^22^
11	Annual surface greening %	0.00	0.0304	0.0286	0.1365	0.1969	0.2263	0.2107	0.2684	0.3001
12	Sequestered CO2 by green matter, ppmv	0.00	19.06	60.43	65.82	76.74	90.77	105.50	121.97	141.95
13	Sea surface temperature rise, °C	0.00	0.15	0.52	0.57	0.61	0.64	0.68	0.74	0.80
14	Observed sea temperature rise, °C	0.00	0.100	0.400	0.500	0.550	0.610	0.620	0.750	---
15	Average land air temperature rise, °C	0.00	0.19	0.71	0.98	1.05	1.12	1.19	1.28	1.39
16	Observed average land air temperature rise, °C	0.00	---	0.40	0.60	0.70	0.90	1.20	1.40	---
17	Sea level rise, glaciers melt only, mm yr^−1^	0.00	0.57	1.23	1.38	1.27	1.23	1.44	1.79	2.16
18	Observed sea level rise, mm yr^−1^	0.00	---	1.3–1.7	1.3–1.7	1.7–2.3	1.7–2.3	1.7–2.3	2.8–3.6	3.64
19	Stratospheric cooling, °C d^−1^	0.00	−0.05	−0.05	−0.24	−0.36	−0.43	−0.42	−0.56	−0.66
20	Observed stratospheric temp. reduction, °C d^−1^	---	---	---	---	−0.44	−0.44	−0.44	−0.44	---
21	Reduction in stratosphere geopotential height, m d^−1^	0.00	−4.90	−4.90	−24.43	−36.43	−43.70	−42.51	−56.80	−67.24
22	Observed reduction, m d^−1^	---	---	---	−33 to −113	−33 to −113	−33 to −113	−33 to −113	---	---

## Data Availability

The data are readily available online by the links provided under references. Observations of changes in climate parameters are provided by IPCC Fifth Assessment Report (AR5), [1], (https://www.ipcc.ch) in chapters 2, 8, and 13. The National Oceanic and Atmospheric Administration (https://gml.noaa.gov/ccgg/trends/data.html), [12], summarizes observed carbon dioxide concentration in the atmosphere. Recent sea level data are published by Horwath et al. (2022), [21].

## Symbols and Abbreviations

C_PA_	Specific heat of dry air, J kg^−1^ °C^−1^
d	A symbol that denotes variation and infinitesimal variation respectively
d^−1^	Per decade
dppmv	Variation in the concentration of carbon dioxide in the atmosphere, parts per million by volume
dQ_A_	Heat transferred from the atmosphere to the surface, J
dQ_D_	Chemical energy produced by deforestation, J
dQ_F_	Chemical energy produced by combustion of fossil fuels, J
dQ_G_	Heat removed by surface greening, J
dQ_GT_	Heat removed by terrestrial greening, J
dQ_GA_	Heat removed by aquatic greening, J
dQ_S_	Total heat exchanged with the surface, J
dQ_U_	Heat lost by the upper atmosphere, J
g	Gravity acceleration, m s^−2^
Exp (x)	Exponential function, equals to e^x^
Γ	Annual precipitation, mm
h	Sea level elevation, mm
kJ	Thousand Joule
L_F_	Ice latent heat of melting, J kg^−1^
L_V_	Latent heat of water evaporation, J kg^−1^
M_A_	Air circulation flow rate at surface, kg yr^−1^
M	Mass of the atmosphere, kg
µ_CO2_	Chemical potential of carbon dioxide, kJ mol^−1^
µ_HO2_	Chemical potential of water vapor, kJ mol^−1^
n	Number of moles or number of years
η	Efficiency of seasonal photosynthesis
η_A_	Efficiency of the atmosphere as a Carnot heat engine cycle
η_max_	Maximum value of the seasonal efficiency of photosynthesis ≈ 2η
n_CO2_	Number of moles of carbon dioxide in the atmosphere, mol
n_H2O_	Number of moles of water vapor in the atmosphere, mol
P	Pressure of the atmosphere, Pa
ppmv	Parts per million by volume
ppmv_CO2_	Concentration of carbon dioxide in the atmosphere in parts per million by volume
Q_C_	Heat rejected to the cold reservoir, atmosphere, J
Q_G_	Biomass inventory, J
Q_G0_	Initial biomass inventory, J
Q_H_	Heat supply by the heat reservoir, surface water, J
H	Enthalpy of ocean and glaciers, J
H_A_	Enthalpy of the atmosphere, J
T_S_	Average surface temperature of the earth, °K.
T_T_	Average temperature of the upper troposphere, °K.
T_L_	Land surface air temperature, °C
U	Internal energy of ocean and glaciers, J
U_T_	Heat transfer coefficient between atmosphere and surface, W m^−2^ °K^−1^
V	Volume of the atmosphere, m^3^
W_A_	Work produced by the atmosphere at average conditions, J
W′_A_	Work produced by the atmosphere with carbon dioxide emission, J
W_S_	Air humidity at saturation with sea temperature, kg water per kg dry air
yr	Abbreviation for year
